# The efficacy of Viscocanalostomies and combined phacoemulsification with Viscocanalostomies in the treatment of patients with glaucoma: a non-randomised observational study

**DOI:** 10.1186/s12886-018-0773-7

**Published:** 2018-05-02

**Authors:** Andrew Want, Derek K.-H. Ho, Bhavani Karri, Divya Mathews

**Affiliations:** Stanley Eye Unit, Abergele Hospital, Llanfair Road, Abergele, Conwy LL22 8DP UK

## Abstract

**Background:**

To evaluate the outcomes of Viscocanalostomy (VC) and Phacoviscocanalostomy (PV) in controlling primary and secondary glaucoma in a large cohort of patients from a single eye unit and performed by a single surgeon.

**Methods:**

This non-randomised, retrospective study was conducted on 620 eyes of 458 patients. All patients who had either viscocanalostomy (VC) or combined phacoemulsification and viscocanalostomy (PV) over a three-year period were included. Intraocular pressures (IOP), number of anti-glaucoma medications used, and any complications were recorded over a 3-year follow up period.

Paired T-Test was used to compare preoperative and post-operative IOP at specified time points. Kaplan–Meier survival models were used to determine success rates over the study period.

**Results:**

Six hundred twenty procedures were performed during the 3-year study period, of which 427 were PV and 193 VC. The mean follow-up was 31.8 months. Overall complete success (IOP ≤ 21 mmHg, without medication) at 3 years was achieved in 65.7% of patients, with qualified success (IOP ≤21 mmHg with or without medication) achieved in 96.0%. Subgroup analysis showed complete success rate of 76.0% for PV and 63.1% for VC (*p* = 0.005), with qualified success 95.9% for PV and 94.0% for VC (*p* = 0.668).

Mean pre-operative IOP (mmHg) for all procedures was 23.02 ± 5.6, with PV and VC subgroups at 22.54 ± 5.10 and 24.06 ± 6.45. Post-operatively IOP at month 12 and 36 was 14.74 ± 3.57 and 14.40 ± 3.17 respectively for all procedures, 14.62 ± 3.26 and 14.44 ± 3.10 for PV, and 15.03 ± 4.18 and 14.31 ± 3.33 for VC.

Across all procedures, pre-operatively an average of 3.05 ± 0.96 anti-glaucoma medications were used. This reduced to 0.13 ± 0.39 in 12 months and 0.38 + 0.71 by 36 months.

Sixty-five cases had complications due to trabeculo-Descemet window perforation during viscocanalostomy with 7 cases developing complications from the cataract element. In the 12.9% of patients who had complications there were no differences of IOP noted at 3 years.

**Conclusion:**

VC and PV have good IOP lowering capacity and are both effective at sustaining a reduction in IOP at 3 years. PV achieved a higher success rate without medication. The low complication profile with reduced post-operative care means these procedures may be a preferred option for early surgical intervention.

## Background

Surgical treatment for glaucoma was traditionally used as a last resort treatment due to the inherent risks associated with the procedures. However the benefit of early surgical intervention is being increasingly recognised, allowing significant long-term reduction of intraocular pressure (IOP) and subsequently slowing the progression of visual field defects. Reducing the need for medical therapy and the subsequent prolonged exposure to topical treatments and preservatives is also beneficial to the ocular surface [1]. Alternatives to traditional trabeculectomy that avoid anterior chamber entry such as viscocanalostomy (VC) were developed, largely as an attempt to reduce sight-threatening complications [2] such as hypotony and its sequelae of choroidal detachment, maculopathy and shallow or flat anterior chamber. Post-operative management following VC is less intensive, requiring fewer hospital visits. As an added benefit, VC can be combined with cataract surgery in phacoviscocanalostomy (PV), reducing the number of surgeries in the target population who often have cataract as co-morbidity.

Systematic review and meta-analysis by Ruilli et al. [3] found that non-penetrating filtering surgeries had lower complication rates, but were not as effective in reducing IOP as trabeculectomy. However there are still relatively few studies available on the long-term outcomes of VC, and some experienced surgeons have achieved extremely good IOP control [4]. Considering the many positive aspects of VC, it could be a preferable option to trabeculectomy for many patients.

In this study we aim to evaluate how the findings of these previous studies can translate in to real world clinical practice by performing a long-term, retrospective assessment of a large number of patients who have undergone VC. Additionally, we will compare the outcomes when the procedure is combined with cataract extraction and intra-ocular lens implantation.

## Methods

### Participant selection

This was a non-randomised observational study including all patients undergoing VC or PV at the Stanley Eye Unit, Abergele Hospital, UK between 09/11/09 and 18/12/12. They were identified from an intranet database retrospectively, in accordance with the tenets of the Declaration of Helsinki. Verbal consent is obtained from all patients for the use of their anonymised clinical data for research purposes within the clinic. Patient-focused posters are also displayed throughout the clinic explaining our use of anonymised data for research undertaken by the department with contact information for anyone requesting further information about our research database. The local research ethics committee (Wales REC 5, Bangor) and local Research and Development committee have approved this research and the consent procedure.

Patients were offered VC if, despite being on maximum medical therapy, they showed evidence of progression of visual field defects on two consecutive Humphreys field assessments, and/or IOP was poorly controlled and out of the target range for the patient. These findings were supported with evidence of corresponding loss of retinal nerve fibre layer (RNFL) and Ganglion cell layer (GCL) on optical coherence tomography (OCT). Patients were also offered surgery if they were intolerant to topical glaucoma medication. The patient’s target IOP was tailored according to age and rate of progression of glaucoma. Patients were excluded if they were aphakic, had silicone oil in the eye, had multiple previous glaucoma procedures or showed evidence of neovascular glaucoma. If cataracts were found on clinical examination at the time of listing, then patients were offered the option of a combined surgery. All patients were included, regardless of glaucoma subtype. A control arm was not included as part of the study.

### Viscocanalostomy and Phacoviscocanalostomy

VC was performed as described in Stegmann et al. [2] A small trabeculo-Descemet window was exposed, thereby avoiding the juxtacanalicular tissue which is the presumed area of resistance. This allows aqueous to percolate through the newly created window allowing decompression of the eye in a controlled fashion. Viscoelastic is injected into the ostia of the Schlemm’s canal thus dilating it and the collector channels. This causes focal microscopic ruptures in Schlemm’s canal endothelial wall and the adjacent juxtacanalicular trabecular membrane. Aqueous can egress via various outflow canals, but ultimately into episcleral vasculature or the uveoscleral route. The intrascleral lake formed by removal of deep scleral tissue allows fluid to escape via the conjunctiva and its lymphatics as well as newly formed aqueous outflow vessels. If the superficial flap developed any button hole, then the excised bit of sclera was used as a patch graft and placed over the superficial flap to avoid bleb formation. The patch graft was secured with 10–0 vicryl. When VC was combined with cataract surgery (PV), the phacoemulsification incision was placed between the superficial and deep flap once the Schlemm’s canal was identified. Phacoemulsification of cataract was completed using a standard approach and then the remainder of the viscocanalostomy was completed. A single surgeon with previous experience of VC and PV performed all procedures.

IOP was measured using Goldmann applanation tonometry (GAT) pre-operatively, and then on postoperative days 1 and 7, week 1, months 1, 3, 6, 9 and 12. Patients were then reviewed every 6 months up to year 3. Measurements were taken during routine outpatient clinics and not at consistent times of day or adjusted for diurnal variations.

The primary outcome of the study was IOP. Secondary outcomes were the number of anti-glaucoma drops used and the frequency of complications. Visual acuity, visual fields and optic nerve head appearance were not assessed.

Success criteria were defined as:

- Complete success: IOP ≤ 21 mmHg ± laser goniopuncture (LGP) and without medication
- Qualified Success: IOP ≤ 21 mmHg ± LGP ± medication

Patients with IOP of ≥22 mmHg, or who underwent repeat glaucoma surgery were noted as surgical failure. These criteria were used to allow comparison with other studies that have used similar end points [4–8]. Kaplan–Meier survival models were used to indicate success rate over the three-year study period, taking censoring into account as some patients transferred to another unit or were lost to follow-up.

The pre- and post-operative IOPs were compared using paired *t* test. A comparison of pre- and post-operative IOPs was made between PV and VC surgeries using unpaired *t* test. The numbers of anti-glaucoma medications used pre- and post-operatively were analysed. Statistical analyses were performed using Microsoft Excel 2013 and SPSS 17.0. A *p* value of < 0.05 was considered statistically significant. Figures were given as mean ± standard deviation (SD).

## Results

### Patient characteristics

A total of 458 patients and 620 eyes were included in the study. 323 left and 297 right eyes were operated upon.

Two hundred twenty-three were male with an age range of 18–93 (74.5 ± 10.9) and 235 were female with an age range of 43–97 (76.7 ± 8.8).

The majority of eyes included were POAG, accounting for 65.0% (403 eyes). The next largest groups were NTG and PXF accounting for 17.10% (106 eyes) and 5.81% (36 eyes) respectively.

Mean follow-up duration was 31.8 (SD 9.3) months. Over 80% of subjects were followed up for the full three-year duration. Patients who had been lost to follow up were not included when calculating mean results. 118 patients had been lost to follow up by the end of the 36 months.

**Fig. 1:**
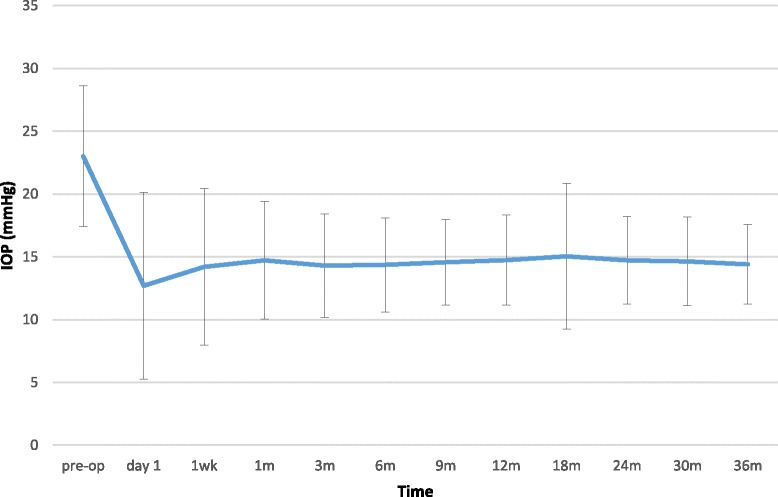
Mean IOP (mmHg) over 36 months for all procedures

The mean pre-operative IOP across all procedures was 23.02 ± 5.60. The mean IOP at the first post-operative day was 12.71 ± 7.42 (*p* < 0.0001). After one month IOP was reduced by 36.0% (from 23.02 mmHg down to 14.73 mmHg). This is similar to the IOP reduction of 37.4% seen by the end of three-year period. Table 1 and Fig. 1 demonstrate that the IOP remained stable throughout follow-up. Complete success rate at 36 months was 65.7% with a qualified success rate of 96.0% (*n* = 502).

**Table 1:** Mean IOP (mmHg) over 36 months for all procedures

Time	pre-op	day 1	1wk	1 m	3 m	6 m	9 m	12 m	18 m	24 m	30 m	36 m
Mean IOP (mmHG)	23.02 ± 5.60	12.71 ± 7.42	14.20 ± 6.24	14.73 ± 4.70	14.29 ± 4.11	14.36 ± 3.74	14.57 ± 3.39	14.74 ± 3.57	15.05 ± 5.80	14.72 ± 3.50	14.64 ± 3.53	14.40 ± 3.17
*P*-value	*p* < 0.001	*p* < 0.001	*p* < 0.001	*p* < 0.001	*p* < 0.001	*p* < 0.001	*p* < 0.001	*p* < 0.001	*p* < 0.001	*p* < 0.001	*p* < 0.001	*p* < 0.001

### PV vs VC

A total of 427 PV surgeries and 193 VC surgeries were performed. A significant IOP reduction of 7.92 mmHg (*p* < 0.0001) was achieved by PV surgery after 12 months, which was maintained up to the end of study period at 36 months. VC patients maintained a similar mean IOP throughout the post-operative follow up period with no significant difference between the groups, but had a larger reduction of 9.03 mmHg (*p* < 0.0001) due to a significantly higher listing mean IOP. Listing mean IOP was 22.54 ± 5.10 for PV and 24.06 ± 6.45 for VC (*p* = 0.0017) (Table 2).

**Table 2:** Mean IOP (mmHg) of VC and PV over 36 months

Time	Pre-operative	12 months	24 months	36 months
No. of eyes PV	427	396	367	356
No. of eyes VC	193	178	157	146
PV mean IOP (mmHg)	22.54 ± 5.10	14.62 ± 3.26	14.81 ± 3.44	14.44 ± 3.10
VC mean IOP (mmHg)	24.06 ± 6.45	15.03 ± 4.18	14.51 ± 3.63	14.31 ± 3.33
	*p = 0.0017	*p = 0.2051	*p = 0.3733	*p = 0.6815

*2-tailed unpaired *t* test

**Fig. 2:**
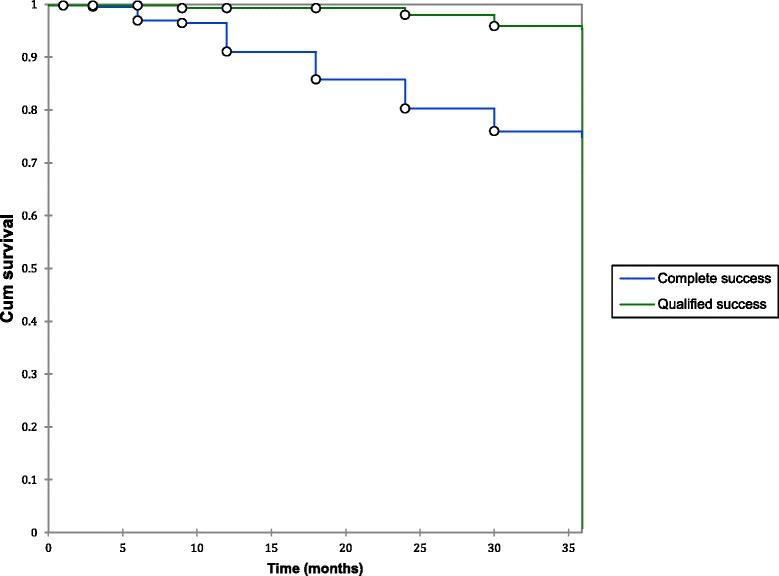
PV survival distribution function

**Fig. 3:**
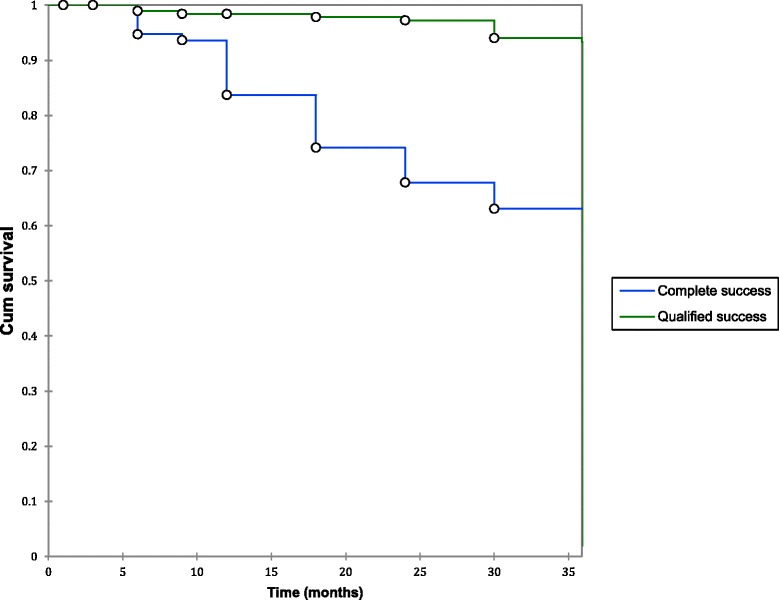
VC survival distribution function

The complete success rate at 36 months for VC was 63.1%, and qualified success rate was 94.0% (*n* = 149). Complete success rates were significantly higher for PV at 76.0% (*p* = 0.0050). There was no significant difference in qualified success with a rate of 95.9% (*n* = 364, *p* = 0.668) (Fig. 2, Fig. 3).

### Anti-glaucoma eyedrops

Throughout the study period, patients were prescribed anti-glaucoma eyedrops as pharmacological intervention to maintain IOP control, at the clinician’s discretion. If a patient was lost to follow up or did not attend an appointment they were not included in the results for that follow up year.

There was a significant reduction in the number of anti-glaucoma drops after surgical intervetnion. Across all procedures, pre-operatively an average of 3.05 ± 0.96 anti-glaucoma medications were used. This reduced to 0.13 ± 0.39 in 12 months, gradually increasing to 0.27 ± 0.59 by 24 months and 0.38 + 0.71 by 36 months. An average reduction of 2.67 medications (*p* < 0.0001) was achieved at the end of the 3 year study period.

At 12 months, 13 patients (2.1%) required anti-glaucoma eyedrops to maintain an IOP of ≤21 mmHg. This increased to 116 (18.7%) and 152 (24.5%) at 24 and 36 months respectively.

**Fig. 4:**
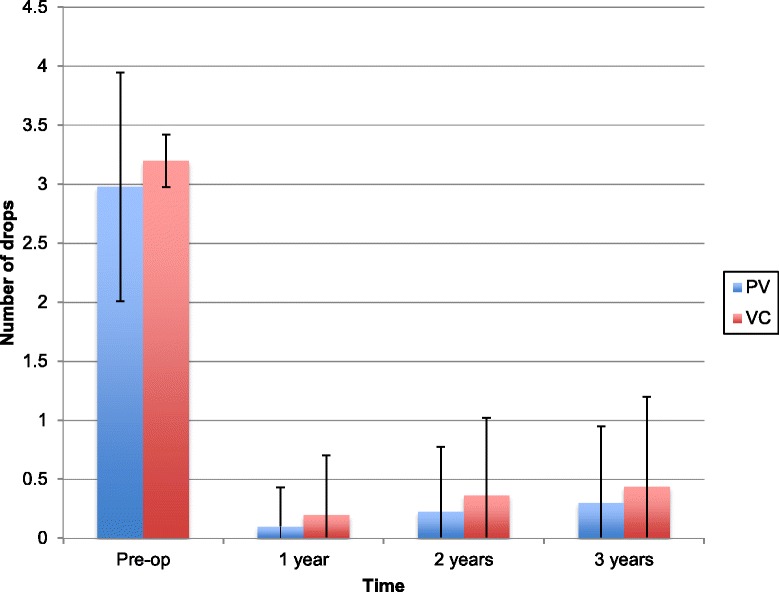
Mean number of anti-glaucoma eye drops

The mean number of drops was higher for the VC group at all points throughout the study (Fig. 4).

### Laser goniopuncture

Outpatient laser goniopuncture (LGP) was performed as a first line treatment in patients who showed increase in IOP and/or visual field progression following VC or PV. Early LGP can be used to create a break in the trabeculo-Descemet membrane window (TDW) and refill a potentially dry scleral lake. LGP was performed in 33.9% (108) of PV patients and 46.21% (61) of VC patients, corresponding to 37.47% (169) of eyes across all procedures with a mean IOP reduction of 23% (6.46 mmHg) from pre-LGP to final follow up. The difference in LGP rate between the PV and VC was not significant (*p* = 0.1120). LGP was required slightly earlier in VC than in PV patients with a mean time of 11.37 months and 14.57 months.

### Complications

We recorded 7 PCR/vitrectomy, 65 perforated TDW and 8 scleral defect patch graft, representing a complication rate of 12.9% across all procedures.

Comparing the post-operative IOP between surgeries with complications and overall results revealed a significant difference in IOP on day 1, where a 2.07 mmHg difference was observed (*p* = 0.0363). At all other time points the difference was not statistically significant (Table 3).

**Table 3:** Mean IOP (mmHg) over time (months) comparing complicated patients to the total study population

Time	Pre-op	day 1	1 m	6 m	12 m	18 m	24 m	36 m
Complicated cases mean IOP (mmHg)	23.03 ± 4.98	10.64 ± 8.63	15.02 ± 4.18	14.38 ± 3.56	14.76 ± 3.42	14.64 ± 3.15	14.44 ± 3.53	14.30 ± 3.82
All cases mean IOP (mmHg)	23.02 ± 5.60	12.71 ± 7.42	14.73 ± 4.70	14.36 ± 3.74	14.74 ± 3.57	15.05 ± 5.80	14.72 ± 3.50	14.40 ± 3.17
*P*-value	*p* = 0.9752	*p* = 0.0363	*p* = 0.5463	*p* = 0.9629	*p* = 0.9777	*p* = 0.6007	*p* = 0.5426	*p* = 0.8353

## Discussion

Previous studies have a found a wide range of complete success rates for VC [3–13], with different criteria for outcomes, length of follow up, patient groups and techniques. Hondur et al.’s meta-analysis [13] reached a final complete success rate of 51.10% at 25.6 months for VC alone. Our patients showed a complete success rate of 65.7% with a qualified success rate of 96.0% at 36 months across all procedures. These outcomes are notably better than the final results of the Hondur et al. meta-analysis [9], however they did not reach the levels of Wishart et al. [4]

The variation of outcomes seen in VC, and other forms of glaucoma surgeries, is thought to be multifactorial. It can be influenced by elements such as variation in individual surgical technique, study population, and the experience of the surgeon. It has previously been reported that approximately 40 cases are needed to master the technique of VC [14]. In our study one surgeon, who had prior experience and familiarity with the technique, performed all procedures.

Listing mean IOP for VC was higher than PV but the post-operative mean IOP’s for both procedures were comparable throughout the follow up period, with no statistically significant difference between the groups. VC and PV both achieved a reduction in IOP and sustained a similar IOP throughout the follow up period. PV did however have a significantly higher complete success rate. We postulate that cataract removal might be a contributory factor to a lower IOP as shown is previous studies [15–17], therefore leading to a higher complete success rate.

The total complication rate for this study was 12.9% with no episodes of hypotony. Cases that developed complications during the procedure had a slightly lower IOP on day 1 post-operatively compared to the rest of the study population. However no significant difference was found across the remainder of the follow up. Overall the complication rates for VC and PV were low, consistent with the findings of previous studies and further supports the position that these procedures are a safer alternative to traditional trabeculectomies [3]. VC and PV also require fewer follow up visits and has simpler post-operative care compared to penetrating surgeries. This reduces the impact on patients’ quality of life as well as reducing the burden on services providers.

### Limitations

There were several limitations of the study. Namely, the study was performed retrospectively, and assessment of patients including IOP monitoring was not masked and therefore subject to observer bias. Visual acuity, visual field assessments and optic nerve head appearance were not used as outcomes of the study, and disease staging was not performed.

IOP was used as the primary outcome. This is an indirect measure of the disease progression but has been used as a justifiable surrogate in similar studies. IOP was measured during routine outpatient clinic appointments and therefore was not measured at a consistent time of day or measured as a mean of diurnal variation.

By including all patients during the time period of the study, multiple subtypes of glaucoma such as NTG were treated. This was because the purpose of the study was to present outcomes in real world practice, however it does create limitations interpreting the data. Especially when comparing the results to other studies, the majority of which limited the study populations to a single subtype such as POAG. Additionally the decision to offer patients surgical treatment was based on the individual patient’s target IOP range. Patients with NTG were therefore offered surgery at a lower IOP.

NTG patients achieved reduction in IOP with sustained control but, as expected, had slightly lower mean IOP when compared to POAG patients. Listing mean IOPs showed the most notable difference to POAG (NTG 18.07 +/− 2.44 mmHg and POAG 23.55 +/− 5.15 mmHg respectively), however this gap narrowed at 36 months (NTG 13.53 +/− 2.24 mmHg and POAG 14.67 +/− 3.37 mmHg). This slightly reduced the overall means, as well as the percentage decrease. It is encouraging though to see that NTG also responded well to the procedures.

Our study did not include a trabeculectomy group or other non-penetrating surgical procedures and therefore direct comparison is not possible. It must be noted however that the available literature directly comparing trabeculectomy to non-penetrating filtration surgeries has consistently found better IOP control in the trabeculectomy groups but with more frequent complications [18–23].

## Conclusion

Our results suggest that VC/PV is an effective treatment for sustainably lowering IOP in glaucoma patients, and reduces the need for IOP-lowering medications. VC and PV have low complication rates and avoid bleb formation and subsequent bleb-related complications, giving these procedures an advantage over other non-penetrating surgeries such as deep sclerectomy. Additionally, combining the procedure with cataract extraction provides good IOP control and avoids additional surgery in future.

Though current evidence suggests VC and PV may not reduce IOP as much as trabeculectomy, their reduced surgical risks mean they may be a more palatable choice than other surgical treatments and can be offered to patients at an earlier stage, and in combination with cataract surgery.

## Abbreviations

GAT: Goldmann applanation tonometry
GCL: Ganglion cell layer
IOL: intraocular lens
IOP: Intraocular pressure
LGP: Laser goniopuncture
NAG: Narrow angle glaucoma
NTG: Normal tension glaucoma
OCT: Optical coherence tomography
OHT: Ocular hypertension
PCR: Posterior capsule rupture
POAG: Primary open angle glaucoma
PV: Combined Phacoemulsification of cataract with intraocular lens insertion/Viscocanalostomy
PXF: Pseudoexfoliation syndrome glaucoma
RNFL: Retinal nerve fibre layer
SD: Standard Deviation
TDW: Trabecular-Descemet’s membrane window
VC: Viscocanalostomy
